# Synergistics of Carboxymethyl Chitosan and Mangosteen Extract as Enhancing Moisturizing, Antioxidant, Antibacterial, and Deodorizing Properties in Emulsion Cream

**DOI:** 10.3390/polym14010178

**Published:** 2022-01-03

**Authors:** Nareekan Chaiwong, Yuthana Phimolsiripol, Pimporn Leelapornpisid, Warintorn Ruksiriwanich, Kittisak Jantanasakulwong, Pornchai Rachtanapun, Phisit Seesuriyachan, Sarana Rose Sommano, Noppol Leksawasdi, Mario J. Simirgiotis, Francisco J. Barba, Winita Punyodom

**Affiliations:** 1 Faculty of Agro-Industry, Chiang Mai University, Chiang Mai 50100, Thailand; meen.nareekan@gmail.com (N.C.); kittisak.jan@cmu.ac.th (K.J.); pornchai.r@cmu.ac.th (P.R.); phisit.s@cmu.ac.th (P.S.); noppol@hotmail.com (N.L.); 2 Cluster of Agro Bio-Circular-Green Industry, Chiang Mai University, Chiang Mai 50100, Thailand; warintorn.ruksiri@cmu.ac.th; 3 Center of Excellence in Materials Science and Technology, Faculty of Science, Chiang Mai University, Chiang Mai 50200, Thailand; winita.punyodom@cmu.ac.th; 4 Faculty of Pharmacy, Chiang Mai University, Chiang Mai 50200, Thailand; pimporn.lee@cmu.ac.th; 5 Faculty of Agriculture, Chiang Mai University, Chiang Mai 50200, Thailand; sarana.s@cmu.ac.th; 6 Faculty of Sciences, Institute of Pharmacy, Universidad Austral de Chile, Valdivia 509000, Chile; mario.simirgiotis@uach.cl; 7 Department of Preventive Medicine and Public Health, Food Science, Toxicology and Forensic Medicine, Faculty of Pharmacy, Universitat de València, Avda. Vicent Andrés Estellés, 46100 Burjassot, València, Spain; francisco.barba@uv.es

**Keywords:** carboxymethyl chitosan, mangosteen, deodorant, skin moisturizing, trans-2-nonenal, accelerated stability test

## Abstract

Carboxymethyl chitosan (CMCH) from native chitosan of high molecular weight (H, 310–375 kDa) was synthesized for improving water solubility. The water solubility of high-molecular-weight carboxymethyl chitosan (H-CMCH) was higher than that of native chitosan by 89%. The application of H-CMCH as enhancing the moisturizer in mangosteen extract deodorant cream was evaluated. Different concentrations of H-CMCH (0.5–2.5%) were investigated in physicochemical characteristics of creams, including appearance, phase separation, pH, and viscosity, by an accelerated stability test. The different degrees of skin moisturizing (DM) on pig skin after applying H-CMCH solution, compared with untreated skin, water, and propylene glycol for 15 and 30 min using a Corneometer^®^, were investigated. The results showed that the 0.5% H-CMCH provided the best DM after applying the solution on pig skin for 30 min. Trans-2-nonenal, as an unsatisfied odor component, was also evaluated against components of the mangosteen extract deodorant cream, which were compared to the standard, epigallocatechin gallate (EGCG). In addition, DPPH and ABTS radical scavenging activity, ferric reducing antioxidant power (FRAP), and antibacterial activities were examined for the mangosteen extract deodorant cream using 0.5% H-CMCH. Results indicated that the mangosteen extract synergized with H-CMCH, which had a good potential as an effective skin moisturizing agent enhancer, deodorizing activity on trans-2-nonenal odor, antioxidant properties, and antibacterial properties.

## 1. Introduction

Emulsions are the most common form of skin care products [1]. A variety of cosmetic emulsions are utilized for functional applications, such as sebum control, skin whitening, and UV protection [2]. Cosmetic emulsions are mainly classified as oil-in-water (O/W) [3], water-in-oil (W/O) [4], or water-in-silicone (W/S) emulsions [5,6]. Emulsions applied in cosmetic applications, both O/W and W/O types, need to satisfy several requirements, such as having the rheology for skin, feeling good on the skin, having good spreadability, and long-term physical stability under various conditions. The ingredients are safe and do not cause skin irritation or any harmful effects [1]; O/W emulsions are the most commonly used in the cosmetic industry. Many cosmetic industries produce a wide variety of beauty products to care for and to avoid excessive sweating and body odor [7]. Deodorants are one of the cosmetic preparations containing substances or ingredients able to eliminate or reduce body odor [8]. Body odor is caused by the growth of microorganisms, and odors are associated with perspiration and its breakdown by bacteria in the armpits, feet, or any other part of the body [9]. Many products claim to have skin benefits, such as anti-aging [10], skin tightening, and moisturizing activity [11]. In general, the preparation of deodorant is carried out using emulsion systems, where the active ingredients are mixed with waxes, oils, and silicones and produced in the desired form, such as deodorant cream, gel, roll-ons, and sticks [12].

Carboxymethyl chitosan (CMCH) is a polymer synthesized by introducing a carboxymethyl group into the main structure of chitosan, achieved by carboxymethylation of the hydroxyl and amine of chitosan, [13] and shows a potential application in cosmetics [14]. The water-soluble property of carboxymethyl chitosan provides conclusive insights into the utilization of its properties of biocompatibility, biodegradation, biological activity, and low toxicity [15]. CMCH in the cosmetics industry seems to be a promising avenue to boost its application as a multifunctional ingredient. Different aspects of CMCH have been applied in five major directions, as a moisture retention agent, antimicrobial agent, antioxidant agent, delivery system, and naturally derived emulsion stabilizer [14]. However, there are many herbs which have antimicrobial properties, this being a primary prerequisite for the development of deodorant formulations.

The use of mangosteen extract (ME) as a raw material to be used as an active ingredient for the preparation of cosmetic products has been evaluated by Ghasemzadeh et al. [16]. It contains the active ingredients xanthones, tannins, and proanthocyanins, which are predominant in mangosteen. In addition, ME has also demonstrated antibacterial properties, reduced acne inflammation, prevented acne, and also contains antioxidants which help to firm the skin and reduce melanin production in the skin, which whitens the skin [17]. ME is made from *Garcinia mangostana* with a standardized solvent extraction process; the product is easy to use and can be mixed with all types of cosmetics [16]. The mangosteen pericarp contains many compounds with outstanding antioxidant, anti-inflammatory and antibacterial properties, especially for the bacteria *Propionibacterium acnes* and *Staphylococcus epidermidis*. The solid waste obtained after extraction of these compounds from the pericarp is also ideal for the production of useful activated carbon [18], while wastewater generated from the extraction process can be efficiently treated with synergistic catalysts [19]. Pothitirat et al. [20] revealed that ME can inhibit the cause of acne and reduce acne rash. Deodorants are substances applied to the body in order to affect body odor caused by bacterial growth and the smell associated with bacterial breakdown of sweat in armpits, feet, and other areas of the body. Ham et al. [21] also reported properties that help in the elimination of body odor. ME is commonly used in various products, such as body cleansing products, soaps, shower creams, facial cleansers, acne treatment products, acne gel, serum, and facial cream [22]. Industries have also been promoted with specially developed synthetic cosmetics as the main ingredient. There are many herbs that have antimicrobial properties, which must be a key factor for improving the deodorant property. Again, herbal formulas require deodorizing properties with activity close to synthetic formulas. However, the synergistic effect of antibacterial activity and deodorizing and moisturizing performance has not been fully investigated. Therefore, this research aimed to determine the effect of CMCH with ME used as moisturizing, antioxidant, antibacterial, and deodorant agents by studying various parameters, then developing final products and evaluating the efficacy of the cream emulsion system.

## 2. Materials and Methods

### 2.1. Materials

High-molecular-weight native chitosan (H, 310–375 kDa) with a degree of deacetylation above 90% was purchased from Kritnarong Limited Partnership, Phitsanulok, Thailand. Ethanol, methanol, isopropanol, sodium hydroxide, and glacial acetic acid were purchased from RCI Labscan (Bangkok, Thailand). Monochloroacetic acid and trans-2-nonenal were obtained from Merck KGaA (Darmstadt, Germany). Aluminium chlorohydrate, ceteareth-25, glyceryl monostearate, propylene glycol, and stearyl alcohol were bought from Thai Poly Chemicals (Samutsakhon, Thailand). All other reagents were of analytical grade. Six species of bacteria: *Corynebacterium* spp. (TISTR 1259), *Staphylococcus epidermidis* (TISTR 518), *Staphylococcus aureus* (ATCC 25923), *Bacillus subtilis* (DMST 15896), *Pseudomonas aeruginosa* (TISTR 781), and *Escherichia coli* (ATCC 25922) were obtained from Thailand Institute of Scientific and Technological Research (Pathum Thani, Thailand).

### 2.2. Synthesis of CMCH

H-CMCH was synthesized following the method of Chaiwong et al. [23]. Chitosan flake was ground and sieved to obtain a particle size under 60 mesh (Endecotts, UK). The chitosan (25 g) was suspended in 50% (*w*/*v*) sodium hydroxide solution (400 mL), and 100 mL of isopropanol was added and mixed well at 50 °C for 1 h. Monochloroacetic acid (50 g) was dissolved in isopropanol (50 mL) and gradually dropped into the reaction for 30 min, and the system was left to stand in reaction at 50 °C for 4 h. The reaction was stopped by adding 70% (*v*/*v*) methanol. The pH of the sample was later adjusted to 7.0 by 1% (*v*/*v*) glacial acetic acid. From that point, the solid was separated and washed with 70% (*v*/*v*) ethanol 5 times, 250 mL each time, and finally washed with 250 mL of 95% (*v*/*v*) ethanol for desalting and dried in a hot air oven (ED56, Binder GmbH, Tuttlingen, Germany) at 80 °C for 12 h. The functional groups of high-MW native chitosan and H-CMCH were measured using a Fourier transform infrared spectrometer (Frontier, PerkinElmer, Waltham, MA, USA) in the range of 500–4000 cm^−1^ as shown in Figure S1. Chitosan was converted to CMCH. The –COO groups enhanced the hydrophilic properties of the CMCH. A certain amount (10 g) of H-CMCH powder was dissolved in 20 mL of deionized water. The suspension was mechanically stirred at 50 °C for 10 min by following the method of Rachtanapun et al. [15] for use as a moisturizing agent.

### 2.3. ME Preparation

Mangosteen fruits were purchased from a local market in Fresh Fruits Market, Chiang Mai, Thailand. Mangosteens with reddish purple skin were selected at the fifth color level according to the Thai Agricultural Standard for Mangosteen (TAS 2-2013) color index [24]. The mangosteen fruits were rinsed with distilled water to remove impurities such as dust before the pericarp was separated from the fruits manually. The mangosteen pericarp was chopped into small pieces and dried at 60 °C. The dried mangosteen pericarp was ground into powder using a blending machine. The powdered mangosteen pericarp (5 g) was thoroughly extracted using a sonication bath (SB25-12 DTD, Ningbo SCIENTZ Biotechnology Co., Ltd., Zhejiang, China) with 50% ethanol (200 mL). The filtrates were concentrated by a rotary evaporator (R-250, Buchi, Flawil, Switzerland) at 50 °C to give a crude extract (200 mg). The ME was kept in air-tight amber bottles and stored at −4 °C until use [20].

### 2.4. Deodorant Cream (O/W) Preparation

Cream base was prepared using an emulsification technique according to the method of Kassakul et al. [25]. First, all ingredients were weighed accurately by a calibrated analytical balance as shown in Table 1. The oil phase was prepared by mixing aluminium chlorohydrate, stearyl alcohol, ceteareth-25, glyceryl monostearate, and mineral oil and then heating to 70 °C. The aqueous phase was prepared by dissolving glycerin and propylene glycol in distilled water in a beaker and heating to 75°C. Both phases (oil and aqueous) were heated up to the same temperature (45 °C) in a water bath; the aqueous phase was added into the oil phase gradually with stirring. Then, ME was added, and different concentrations of H-CMCH, including 0.5, 1.0, 1.5, 2.0, and 2.5% (H1, H2, H3, H4, and H5) were added into the cream base. Finally, the weight of cream was 100 g, and 0.01 mL of perfume was added into the mixture with continuous stirring for 20 min until the cream cooled to 25 °C.

### 2.5. Degree of Skin Moisturizing (DM)

The degree of skin moisturizing of the deodorant cream was examined with 0.5, 1.0, 1.5, 2.0, and 2.5% (*w/v*) of H-CMCH on pig skin and compared with untreated skin, water, and propylene glycol. The pig skins were prepared from the back side of the pig ear obtained from local market sources (Chiang Mai, Thailand). The samples were washed and cleaned, with removal of the fat layer, prior to cutting into 3 × 3 cm pieces. Each sample (100 µL) was applied to the skin surface. The skin without any substance was used as a control. Skin moisturizing was measured before application to samples and after application at 0, 15, and 30 min intervals using a Corneometer^®^ (Courage-Khazaka Electronic GmbH, Cologne, Germany). Before applying the sample and recording the parameter, the pig skins were kept at 25 °C for 30 min. This method was adapted from Kassakul et al. [25]. The degree of skin moisturizing (%) was tested in triplicate to detect random error and calculated using Equation (1).

(1) $$\mathrm{DM}\;(\%)=\frac{Mi-\;Ma}{Mi}\;\times \;100$$

where *M*_i_ is the initial moisturizing content before the sample was applied to the skin and *M*_a_ is moisturizing content after the sample was applied to the skin.

### 2.6. Accelerated Stability Study

The deodorant creams with different concentrations of H-CMCH (0.5, 1.0, 1.5, 2.0, and 2.5% (*w*/*v*)) were centrifuged (Universal 320R, Hettich, Tuttlingen, Germany) at 6000× *g* for 20 min. Accelerated stability tests were performed at both 4 °C and 45 °C for 24 h in an Incucell incubator (MMM Medcenter Einrichtungen GmbH, München, Germany) for 6 cycles. The physicochemical characteristics of the creams—including visual appearance, pH using a pH meter (FiveEasy F20, Mettler Toledo, Greifensee, Switzerland), viscosity using a Brookfield viscometer (DV-II+, Brookfield, Middleboro, MA, USA), and color L*, a*, b* using a colorimeter (CR-410, Konica-Minolta, Tokyo, Japan)—were monitored at every cycle. Total color difference (∆E) was calculated for each sample and each cycle following the strategy of Tkacz et al. [26].

### 2.7. Deodorizing Activity

The deodorizing activity of the developed deodorant cream was evaluated against the odor component trans-2-nonenal, following the method of Ham et al. [21] by using solid-phase microextraction and gas chromatography. The dilute deodorant cream solutions of different concentrations (1–100 mg/mL) were prepared by dissolving the extract in 0.2 M potassium phosphate buffer solution (pH 7.4). An aliquot (1 mL) of the dilute deodorant cream solutions was mixed with aqueous solution (100 µL) containing the odor compound: 10 ppm of trans-2-nonenal or solution. The mixture of deodorant cream and odor substance was placed in a vial (20 mL), which was tightly sealed with a cap furnished with PTFE/silicone septa (Supelco, Bellefonte, PA, USA). The sample vial was then placed in a stirring water bath at 35 °C for 10 min to achieve phase equilibrium, and then the odor substance in the headspace of the vial was taken by a SPME fiber during additional stirring for 5 min at 37 °C. Carboxen/polydimethylsiloxane (Carboxen/PDMS; 75 µm film thickness) was used for detecting trans-2-nonenal. After the adsorption of the odor substance, the fiber was removed from the vials and immediately inserted into the injector of a gas chromatography system for quantitative analysis. The odor compounds were desorbed from the fiber by heating at 250 °C for 2 min in the gas chromatography system.

Gas chromatography was carried out using a gas chromatography flame ionization detector (GC-2010 Series, Shimadzu, Santa Clara, CA, USA) equipped with a flame ionization detection system. The oven temperature for trans-2-nonenal analysis was programmed at 50 °C for 2 min, from 50 °C to 200 °C at a heating rate of 8 °C/min, 200 °C for 2 min, and finally 250 °C hold for 1 min. Injector and detector temperatures for the analysis of trans-2-nonenal were also 250 °C. The samples were injected in a spitless mode using nitrogen as the carrier gas (1 mL/min) at a volume of 1.0 µL. Deodorizing activity (%) was calculated by Equation (2).

(2) $$\mathrm{Deodorizing}\;\mathrm{activity}\;(\%)=\frac{Hn\;-\;Hc}{Hn}\;\times \;100$$

where *H*_n_ is the headspace amount of the odor substance (trans-2-nonenal) and *H*_c_ is the headspace amount of the deodorant cream.

### 2.8. Antioxidant Properties

The optimal cream formula was selected based on skin moisturizing and deodorizing properties and a stability test for antioxidant activity. These properties were compared in the prototype cream formula (no ME and H-CMCH). Solutions of the developed deodorant cream (stock: 5 mg/mL in distilled water) at different concentrations of 1, 2, 3, 4, and 5 mg/mL were prepared and used for DPPH, ABTS, and FRAP assays.

#### 2.8.1. DPPH Radical Scavenging Activity

The ability of antioxidants to scavenge the 2,2-diphenyl-1-picrylhydrazyl (DPPH) free radical was tested by using the modified method of Surin et al. [27] and Phimolsiripol et al. [28]. After that, 100 µL of the stock samples (as described above, concentrations: 1, 2, 3, 4, and 5 mg/mL) were blended with 100 µL of 0.2 mM DPPH reagent (Sigma-Aldrich, Singapore) and incubated at 25 °C for 30 min in the dark. Absorbance was measured at 517 nm in a 96-well microplate reader (SpectraMax^®^ i3x, Molecular Devices, San Jose, CA, USA). The radical scavenging activity of the sample was calculated based on gallic acid (Sigma-Aldrich, Darmstadt, Germany). Results were expressed as milligram gallic equivalent per gram of sample (mgGAE/g sample). The percentage of DPPH radical scavenging activity can be calculated as shown in Equation (3) before plotting of IC_50_ against respective concentration.

(3) $$\%\;\mathrm{DPPH}\;\mathrm{radical}\;\mathrm{inhibition}=\frac{Ac\;-\;As}{Ac}\;\times \;100$$

where *A*_c_ is absorbance of the DPPH solution and *A*_s_ is absorbance of different concentrations of samples.

#### 2.8.2. ABTS Radical Scavenging Activity

2,2-azino-bis-(3-ethylbenzothiazoline-6-sulfonic acid) (ABTS) radical scavenging activity was tested according to the method described by Surin et al. [27,29] and Ruksiriwanich et al. [30]. ABTS (Sigma-Aldrich, Singapore) reagent solution was freshly prepared by mixing 7 mM of ABTS solution with 2.45 mM of potassium persulfate (Sigma-Aldrich, Singapore). ABTS powder and potassium persulfate powder were individually dissolved in water to the required concentration and then combined in a bottle. After 16 h of incubation in the dark at 25 °C, the resultant dark blue color of the ABTS reagent solution was diluted with ethanol until the absorbance reading reached 0.7 ± 0.2. The solutions of H-CMCH were prepared as described previously in 2.6. Each sample solution (0.5 mL at concentrations: 1, 2, 3, 4, and 5 mg/mL) was mixed with 1.0 mL of ABTS stock solution and incubated for 6 min in the dark. Absorbance was measured at 734 nm in the 96-well microplate reader. The ABTS radical scavenging activity was expressed as milligram gallic equivalent per gram of sample (mgGAE/g sample). The percentage of ABTS radical scavenging activity can be calculated as shown in Equation (4) with plotting of IC_50_ against respective concentration.

(4) $$\%\;\mathrm{ABTS}\;\mathrm{radical}\;\mathrm{inhibition}=\frac{Ac\;-\;As}{Ac}\;\times \;100$$

where *A*_c_ is absorbance of the ABTS solution and *A*_s_ is absorbance of different concentrations of samples.

#### 2.8.3. Ferric Reducing Antioxidant Power (FRAP)

The ferric reducing antioxidant power (FRAP) assay was carried out according to the technique of Surin et al. [31]. The FRAP reagent was prepared by mixing 25 mL of 0.3 M acetate buffer (pH 3.6), 2.5 mL of 4,6-tripyridyl-s-triazine (TPTZ) (Sigma-Aldrich, Darmstadt, Germany) solution in 40 mM HCl (RCI Labscan, Bangkok, Thailand), and 2.5 mL of 20 mM ferrous sulphate (Loba Chemie, Mumbai, India). Then, 50 µL of sample (5 mg/mL) was mixed with 950 µL of FRAP reagent and incubated in the dark for 30 min. Absorbance was measured at 593 nm in a 96-well microplate. The ferric reducing antioxidant power of the sample was determined based on ferrous sulphate (Merck KGaA, Darmstadt, Germany). Results were expressed as ferrous sulphate equivalent antioxidant capacity (FEAC) with µmol Fe^2+^/g sample.

### 2.9. Antibacterial Properties

The antibacterial properties on six species of bacteria—*Corynebacterium* spp., *S. epidermidis*, *S. aureus*, *B. subtilis*, *P. aeruginosa*, and *E. coli*—were tested using the agar well diffusion method by Bai-Ngew et al. [32]. For this step, a good representative formula was compared with the prototype cream formula (no ME and H-CMCH). The bacterial culture was swabbed on sterile nutrient agar plates. Subsequently, filter paper discs (6 mm in diameter) were dipped into the prototype deodorant cream, developed deodorant cream (stock 5 mg/mL in distilled water), and positive control (10 mg/mL Streptomycin). The plates were incubated at 37 °C for 18–24 h in an upright position. The experiment was carried out in triplicate, and the inhibition zone was recorded and expressed in millimeters.

### 2.10. Statistical Analysis

All data were analyzed by one-way ANOVA. Mean comparison was performed by Duncan’s multiple range tests with significance level *p* < 0.05. Statistical analyses were performed with the SPSS 17.0 (SPSS Inc.; IBM Corp.; Chicago, IL, USA).

## 3. Results and Discussion

### 3.1. Effect of H-CMCH Synthesis

The yield, moisture content, water solubility, viscosity, and pH of H-CMCH were 45.36%, 5.56%, 89.5%, 360 cP, and 7.33, respectively. Solubility is a significant property of CMCH that measures its resistance to water. The H-CMCH improved water solubility by about 89% when compared to chitosan. The solubility and conformation of CMCH are a result of the deacetylation, pH, and MW of native chitosan. The solubilization process of CMCH is related to functionalized polymers and different types of chemical and physical interactions, such as hydrogen bonds, hydrophobic interactions, and van der Waals forces. High water solubility suggests that CMCH is moisture absorbent and has a greater ability to bind with water than chitosan. However, the solubility of chitosan was relatively poor in water and organic solvents, which resulted in limitation in its uses. At pH < 6.0, chitosan is positively charged (–NH_3_^+^), with increased solubility in water. As the pH increases, chitosan loses its charges, due to protonation, while the amino groups decrease as the solutes begin to precipitate. Chemical modification of amino groups and hydroxyl groups using a carboxymethylation reaction results in a large number of water-soluble chitosan derivatives [14]. This causes the increase of hydrated water molecules around the chains of CMCH than surround the chitosan chains, resulting in higher water solubility. The results are also consistent with the report of Siahaan et al. [33], who found that temperature and NaOH concentration affected CMCH synthesis, and Rachtanapun et al. [34] in carboxymethyl bacterial cellulose. The interactions between NaOH and monochloroacetic acid resulted in reduced CMCH forming and lower solubility. The mitigation in solubility might stem from the loss of free amino functional groups that enhance the hydrophobic nature of the compounds [35]. The greater solubility of L-CMCH and M-CMCH resulted in a decrease in viscosity, but H-CMCH showed higher viscosity. This could be explained as follows, CMCHs with longer chains or higher MW were contributing to the gel. H-CMCH is an effective water-soluble polymer with high viscosity which could be successfully utilized in pharmaceuticals and cosmetics as an emulsion stabilizer and thickening agent. Thanakkasaranee et al. [36] also reported that the yield of CMCH was also dependent on the concentration of NaOH, MW of chitosan, solvent, and reaction temperature. In addition, the solvent ratio and the processing also affect the yield and antioxidant activities [37].

### 3.2. Degree of Skin Moisturizing (% DM)

**Figure 1:**
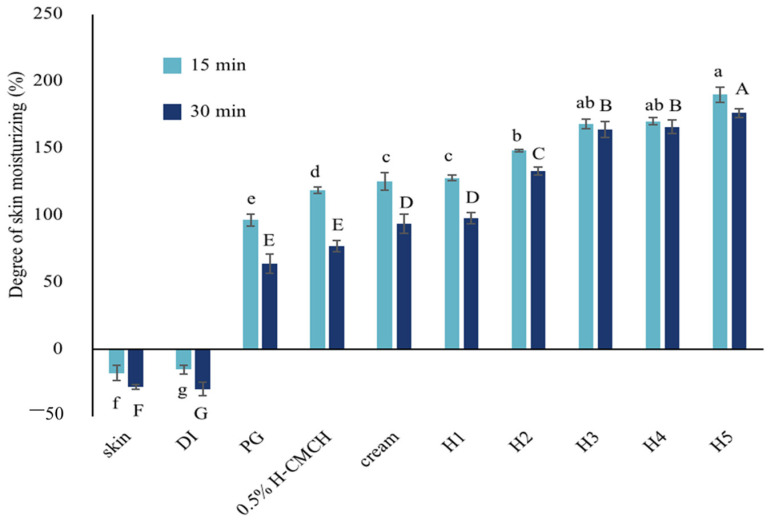
DM (%) as affected by time (15 and 30 min) and different treatments (skin, DI, PG, H1, H2, H3, H4, and H5) on pig skin. Different lowercase letters (a,b,c...) indicate significant differences between solutions at 15 min and different uppercase letters (A,B,C...) indicate significant differences between solutions at 30 min.

The degree of skin moisturizing indicates the water-holding capacity of the skin, which can be tested by the Corneometer^®^ method. The Corneometer^®^ measures the changes in electrical capacitance related to the moisture content of the skin before and after applying the solutions [25]. The degree of skin moisturizing of the deodorant cream with different concentrations of H-CMCH (0.5, 1.0, 1.5, 2.0, and 2.5% (*w*/*v*)) was examined on pig skin and compared with untreated skin, water, and propylene glycol at 15 and 30 min as presented in Figure 1.

The degree of moisturizing at time 15 and 30 min showed that the degree of skin moisturizing of the solutions decreased with increasing time after applying solutions. At the same time, the degree of skin moisturizing of all treatments showed a significant difference after applying between 15 and 30 min (*p* < 0.05). Applying H-CMCH solution for 15 min gave a higher degree of skin moisturizing than 30 min, showing the degree of skin moisturizing of untreated skin, water, propylene glycol, H1, H2, H3, H4, and H5 solutions applied on pig skin for 30 min were significantly decreased compared to 15 min. This confirms that the H-CMCH solution provided a good moisture absorption. In fact, the skin moisturizing effects appeared to decrease with increasing time due to a lack of mechanisms to maintain skin moisturizing and the dryness of pig skin cells [38]. The higher MW CMCH also had superior moisture retention capacity. Kassakul et al. [25] found that 0.2% *Hibiscus rosa-sinensis* mucilage as a natural ingredient provided good results for skin moisturizing after applying for 30 min, improving by about 130%. The results showed that moisturizing products could increase the water content of the skin while maintaining softness and smoothness [39]. Chaiwong et al. [23] reported, after applying solutions containing different MW of water-soluble CMCH (L-CMCH, M-CMCH, H-CMCH), that the moisture content of the skin increased. The mechanism of the moisturizing effect is based on the formation of a water film on the skin surface after dissolution of CMCH, and a subsequent stage of water evaporation could further prevent water evaporation from the skin [40]. Positive electrical charges and relatively high MW facilitate prolonged skin adherence [14]. Our results also showed that H-CMCH decreased the loss of water while elevating skin humidity. The higher apparent viscosity of H-CMCH can improve stability and enhance skin hydration. In fact, H-CMCH was superior to untreated skin, water, and propylene glycol in terms of degree of skin moisturizing effect. The higher concentrations of H-CMCH also indicated potential for film forming and multilayer coating of the skin. Subsequently, it could be used in cosmetic preparations, with further studies suggested to test skin irritation in human subjects.

### 3.3. Effect of Accelerated Stability Study

#### 3.3.1. Visual Appearance

**Figure 2:**
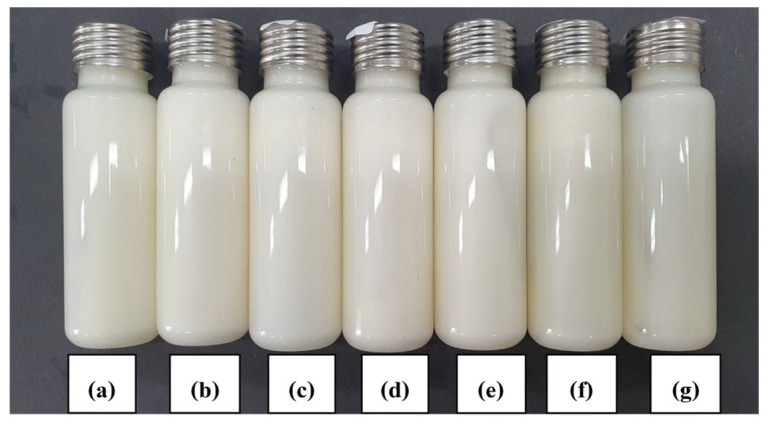
Deodorant cream with 0.5% H-CMCH by heating/cooling cycle; (**a**) cycle 0, (**b**) cycle 1, (**c**) cycle 2, (**d**) cycle 3, (**e**) cycle 4, (**f**) cycle 5, and (**g**) cycle 6.

**Figure 3:**
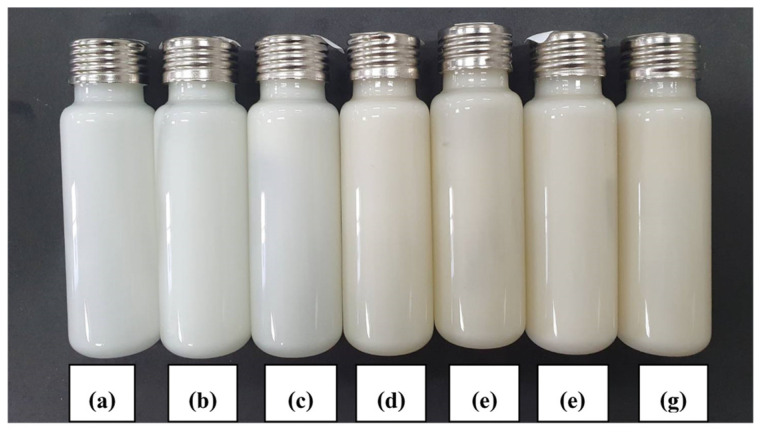
Deodorant cream with 1.0% H-CMCH by heating/cooling cycle; (**a**) cycle 0, (**b**) cycle 1, (**c**) cycle 2, (**d**) cycle 3, (**e**) cycle 4, (**f**) cycle 5, and (**g**) cycle 6.

**Figure 4:**
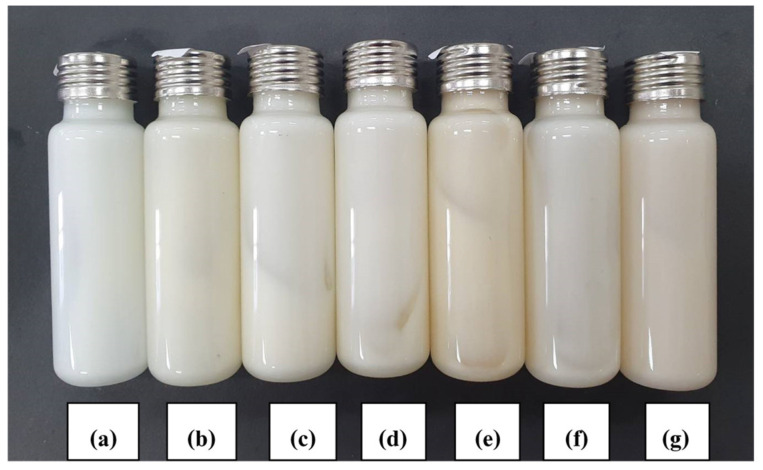
Deodorant cream with 1.5% H-CMCH by heating/cooling cycle; (**a**) cycle 0, (**b**) cycle 1, (**c**) cycle 2, (**d**) cycle 3, (**e**) cycle 4, (**f**) cycle 5, and (**g**) cycle 6.

**Figure 5:**
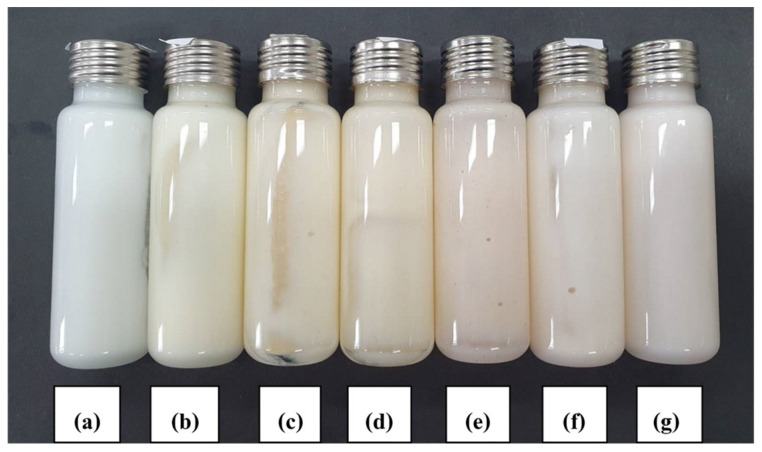
Deodorant cream with 2.0% H-CMCH by heating/cooling cycle; (**a**) cycle 0, (**b**) cycle 1, (**c**) cycle 2, (**d**) cycle 3, (**e**) cycle 4, (**f**) cycle 5, and (**g**) cycle 6.

**Figure 6:**
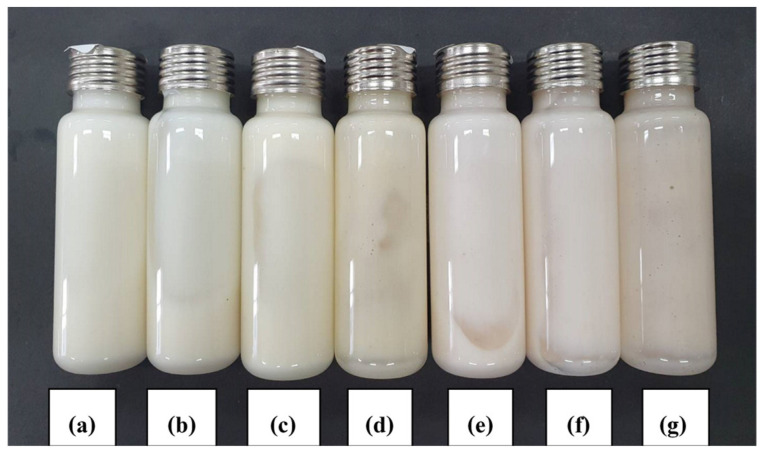
Deodorant cream with 2.5% H-CMCH by heating/cooling cycle; (**a**) cycle 0, (**b**) cycle 1, (**c**) cycle 2, (**d**) cycle 3, (**e**) cycle 4, (**f**) cycle 5, and (**g**) cycle 6.

A heating/cooling cycle test was performed while the formulations were stored in an incubator. The temperature was alternated between 4 °C and 40 °C every 24 h during any period of time. This method is commonly used during the initial stage of developmental screening. Useful information relating to stability could be obtained from such tests [1]. The accelerated stability test of the deodorant cream with different concentrations of H-CMCH (0.5, 1.0, 1.5, 2.0, and 2.5% (*w*/*v*)) was performed at 4 °C in a refrigerator and 45 °C in a hot oven and a heating/cooling cycle for up to 6 cycles. The result of each formulation was randomly checked every 1 cycle by centrifugation at 6000× *g* for 20 min at 25 °C. Deodorant creams with 0.5 and 1.0% H-CMCH indicated acceptable stability after 6 cycles by less phase separation. For the cream containing 1.5–2.5% H-CMCH, higher phase separation and greater changes in color are evident, as shown in Figure 2, Figure 3, Figure 4, Figure 5 and Figure 6.

#### 3.3.2. pH Value

The measurement of pH for all five deodorant formulas in the accelerated state with the heating/cooling cycle method, at the 0 cycle of the stability study the developed deodorant cream samples had pH in the range of 6.32–6.41. After the end of the test, the pH values of all five formulas of deodorant products were found to be in the range of 6.26 to 6.37. This was similar to the pH of human skin and thus suitable for application to the skin with a good stability; for example, the cream texture was a fine opaque white. While applying, the consistency of the cream spreads well; it is easy to blend and can be absorbed into the skin and enhance the moisture of skin for a long time. Moreover, an effective cream should have a pH of about 6.55 [40]. After acceleration to cycle 6, it was found that the pH value decreased significantly (*p* < 0.05), with the pH value in the range of 6.34–6.37 as shown in Figure S2. The pH range should not be too acidic or alkaline, because irritation to the skin might be unavoidable. The pH mitigation is probably due to separation of the cream emulsion from its matrix and ionization, resulting in net negative charge and causing the rise in acidity [41,42]. To avoid exceedingly high pH values beyond the skin’s physiological range, a preservative solution was added to the formulations. Implementation of sodium benzoate or sodium salt of benzoic acid also facilitates stabilization of the skin’s pH. In general, sodium benzoate is commonly used in combination with antiseptics as food preservatives, cosmetics, and medicines [42]. The oxidative/reductive mechanisms, deactivating properties, and safety assessment of benzoic acid or related compounds in biological systems are well documented in the literature [43,44,45]. Considering the kinetic rate (*k*) of pH, it was found that increasing CMCH content (0.5–2.5%) in the formula affected the decrease of the pH’s *k*-value when compared to the formula with 0.5% (*w*/*v*) CMCH. It was evident that cream containing 0.5% (*w*/*v*) H-CMCH showed a good pH stability, which plateaued after six cycles. For other samples, the pH decrease was still ongoing in a linear trend after six cycles. This is probably due to the implementation of higher H-CMCH concentration.

#### 3.3.3. Viscosity

Viscosity is one of key parameters indicating cream quality. The forecasting of this parameter is commonly performed in accelerated stability testing [1]. From the viscosity stability of the deodorant cream after passing the accelerated state for up to six cycles, the results showed that H1 and H2 were not separated and precipitated; the cream texture had a smooth appearance. The viscosity was not significantly changed (*p* ≥ 0.05), being in the ranges 234–300 and 231–313 cP, respectively. H3, H4, and H5 started to separate. The viscosity was significantly decreased (*p* < 0.05), with values in the ranges 154–363, 125–464, and 114–500 cP, respectively, as shown in Figure S3. As for the viscosity changing rate (*k*), as the CMCH content increased from 0.5% to 2.5% (*w*/*v*), the *k*-value increased (from 9.9 to 71.6 cP/cycle). The increasing CMCH content could have affected the decrease in the viscosity value and stability under changing temperature. CMCH is an amphiprotic ether of chitosan derivative. The functional groups include active hydroxyl (–OH), carboxyl (–COOH), and amine (–NH_2_) in the molecule. CMCH is soluble in water at neutral pH (pH = 7). It also exhibits high viscosity as well as film and gel forming capability, which encourages its use in foods and cosmetics [14]. These are excellent properties for work as stabilizers in emulsion preparation [14]. Chaiwong et al. [23] have reported that the greater solubility also corresponded to the decrease in viscosity of the low- and medium-molecular-weight CMCHs, which are slightly different, but for the high-MW CMCH, it required significantly higher viscosity. This could be explained by the fact that CMCHs with chains longer or higher in MW were contributing to the gel. Moreover, it also has been pointed out by Tzaneva et al. [46] that with increasing temperature of emulsions, viscosity and shear stress decreased with different gradients. Using CMCH as a stabilizing agent indicates the ability of its rheological characteristics. After measurement of thermophysical properties by TGA/DTA analysis, it can be concluded that CMCH is suitable to work in the heating process and sterilization at temperatures up to 220 °C without changing the quality of components. The emulsions containing 0.3–0.5% (*w*/*v*) of CMCH could be applied in terms of pharmaceutical and cosmetic oil/water emulsions.3.3.4. Color L*, a*, b* and ∆E.

Color measurements with the colorimeter of deodorant creams with concentrations of 0.5, 1.0, 1.5, 2.0, and 2.5% (*w*/*v*) H-CMCH (H1, H2, H3, H4, and H5) were carried out during the accelerated stability test under 4 °C in a refrigerator and 45 °C in an incubator by heating/cooling cycle (4 °C, 24 h and 45 °C, 24 h) for six cycles. Defined by the Commission Internationale de l’Eclairage (CIE), the L*, a*, and b* color space was modeled after a color-opponent theory. As L* indicates lightness, a* is the red/green coordinate, and b* is the yellow/blue coordinate. The results showed that all five formulas of deodorant cream have an initial L* value (cycle 0) in the range of 79.63–80.07. Moreover, it was found that as the number of cycles of the acceleration test increased, the brightness of five deodorant formulas was significantly reduced (*p* < 0.05), as shown in Figure S4. The H4 and H5 deodorant creams had the lowest L* values compared with H1, H2, and H3 at accelerated cycles 3–6. The separation is caused by high-speed centrifugation, which can accelerate the emulsion precipitation. A good emulsion must withstand a long centrifugal force of 5000–10,000× *g* for 30 min without separation. Shaking or stirring causes more particles in the emulsion to be more mixed. Moreover, reducing the viscosity accelerates the integration of the internal or dispersed phase. This acceleration is achieved by continuously centrifuging the emulsion. Normally, the emulsion stability limited by agglomeration, sedimentation, viscosity of the aqueous phase and rheological properties of the emulsion [47]. This is a result of disintegration or changes in the structure of important substances in the ME and H-CMCH.

For a* (red/green coordinate) values, the result is shown in Figure S5 when considering each formula of deodorant cream during the accelerated stability test for six cycles. It was found that the a* tended to increase in cycle 2 and tended to decrease in cycle 3 until the end of storage. For each cycle in accelerated storage, the results showed that the a* value of the cream deodorant formulas H1, H2, and H3 in cycles 3–6 were in steady decline (*p* ≥ 0.05), ranging from 1.41 to 1.43. Due to the instability of deodorant cream with poor emulsion and lower smoothness, it was clearly seen that the consistency of the cream changed as the number of stability tests increased.

For b* (yellow/blue coordinate) values, the result is shown in Figure S6 when considering each formula of deodorant cream during the accelerated stability test for six cycles. It was found that there was a tendency of the b* value of the deodorant creams to increase from the initial cycle (cycle 0) in the range of 2.1–2.5. For H4 and H5, the b* increased from cycle 1 until the end; the values remained in the ranges 3.26–4.34 and 3.49–4.34, respectively. Meanwhile, b* values for H2 and H3 tended to increase in cycle 2 and gradually remained constant until cycle 2–6 retention, ranging from 2.53–2.93. and 2.54–3.93. For H1, the b* value changed at accelerated cycle 3 until the end of storage (cycles 2–6), being in the range of 2.63–3.43. This showed that increasing storage time had the effect of increasing the b* value of deodorant creams (*p* < 0.05).

**Figure 7:**
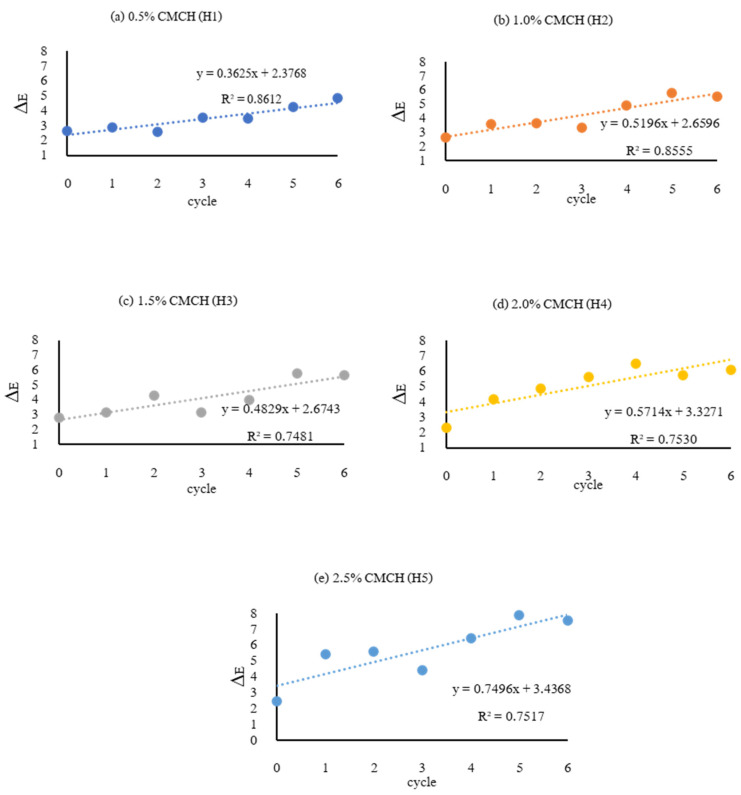
Total color difference (∆E) of deodorant cream adding (**a**) 0.5%, (**b**) 1.0 %, (**c**) 1.5%, (**d**) 2.0% and (**e**) 2.5% (*w*/*v*) H-CMCH; heating/cooling cycle for up to 6 cycles.

The total color difference (∆E) describes incorporated changes in the qualities of L*, a*, and b* through the square root of the sum of square differences between two sets of complete color values [48]. The ∆E of deodorant creams with different concentrations of H-CMCH (0.5–2.5%) was measured during an accelerated stability test performed at 4 °C in a refrigerator and 45 °C in an incubator by heating/cooling cycle (4 °C and 45 °C for 24 h) for six cycles and compared with the basic formula deodorant cream, measuring with the CIE system colorimeter and calculating in the form of ∆E as in Figure 7. In comparison with the white basic deodorant cream, an effect on ∆E resulted. As the development of the deodorant cream involved adding mangosteen extract for the deodorizing agent, the initial color of all five deodorant formulas was white and pale yellow. This could be clearly observed with ∆E in the range of 2.65–3.06. However, as the retention period increased, the results showed that the stability of the cream changed, with visible separation occurring and unstable color, affecting the ∆E, which tended to increase significantly (*p* < 0.05). However, their colors were still acceptable by consumers if the ∆E values were less than 5 [49].

### 3.4. Deodorizing Activity

**Figure 8:**
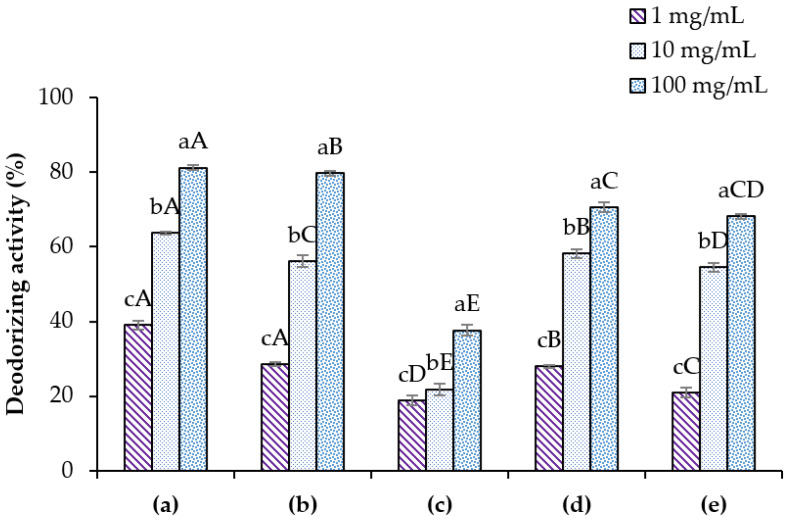
Deodorizing activity of (**a**) ME, (**b**) EGCG, (**c**) prototype cream, (**d**) developed deodorant cream mixed with ME and 1.0% (*w*/*v*) H-CMCH (H2), and (**e**) prototype cream mixed with EGCG and 1.0% (*w*/*v*) H-CMCH at different concentrations (1, 10, and 100 mg/mL). Different lowercase letters (a,b,c...) indicate significant differences between concentrations in the same formula, and different uppercase letters (A,B,C...) indicate significant differences between formulas at the same concentration.

Trans-2-nonenal is an unsaturated aldehyde produced from lipid oxidation, which generates an unpleasant greasy odor. It is known to be a major odor component detected from the bodies of old people [20]. Different concentrations (1, 10, and 100 mg/mL) of each sample—(a) ME, (b) standard EGCG, (c) prototype cream, (d) developed deodorant cream mixed ME and 1.0% H-CMCH (H2), and (e) prototype cream mixed with EGCG standard and 1.0% H-CMCH—were used for deodorizing activity against trans-2-nonenal as shown in Figure 8. It was found that the basic deodorant had the lowest deodorizing activity (18–37%). The deodorizing activity was significantly increased (*p* < 0.05) when ME and EGCG were added to the basic formula deodorant. However, the samples of deodorant cream with ME added and formula with EGCG added at a concentration of 1–100 mg/mL. The results showed that the deodorizing activities were in the range of 27–70% and 21–68%, respectively, which was slightly lower than ME and EGCG standards. The basic formula deodorant contains waxes and fatty acids (fatty acids or fatty alcohol), which are of high MW, high viscosity, non-volatile, and have skin moisturizing properties (by reducing the evaporation of water), but it has no deodorizing properties [50]. Therefore, for some types of deodorant creams or cosmetics, it is imperative to add an active substance to the product in order to increase its antioxidant properties and deodorizing activity.

### 3.5. Antioxidant Properties

The developed formula (ME + 1% (*w*/*v*) H-CMCH) was selected from former experiments in order to compare the antioxidant activities to the prototype formula (no ME and H-CMCH) as presented in Table 2. The developed formula had strong antioxidant activity. Although the DPPH values of the two formulas were not statistically different (*p* ≥ 0.05), the developed deodorant cream showed the greater ABTS values and had higher ferric ion reducing antioxidant power than the prototype formula.

### 3.6. Antibacterial Properties

For the antibacterial properties, the deodorant cream with the mixture of ME and 1% (*w*/*v*) H-CMCH was compared with a basic formula deodorant cream and streptomycin. It was found that the developed deodorant cream could inhibit all six types of bacteria, including *S. aureus*, *S. epidermidis*, *Corynebacterium* spp., *B. subtilis*, *P. aeruginosa*, and *E. coli*, and it was more effective in antibacterial activity than the basic formula (without ME and H-CMCH), as reflected by a greater inhibition zone (Table 3). Table 3 showed that the incorporation of ME and H-CMCH improved the antimicrobial properties of the deodorant cream. Janardhanan et al. [51] reported that mangosteen pericarp extract is known for its antibacterial activity against several pathogens that cause skin infection and acne. Moreover, He et al. [52] prepared the CMCH/lincomycin hydrogels for investigation into antibacterial properties. The antibacterial activities of the hydrogels were tested against Gram-negative *(E. coli*) and Gram-positive (*S. aureus*) bacteria. The result showed that the CMCH/lincomycin hydrogel was expected to be used as an antibacterial agent. Mohamed and Sabaa [53] studied CMCH/silver nanoparticle (Ag) hydrogels with high antibacterial activity against three Gram-positive bacteria (*S. aureus*, *B. subtilis*, and *Streptococcus faecalis*), three Gram-negative bacteria (*E. coli*, *P. aeruginosa*, and *Neisseria gonorrhoeae*), and *Candida albicans* fungus. The hydrophobicity and antibacterial properties of the solid surface are closely correlated with adhesion forces [54].

## 4. Conclusions

H-CMCH showed to be an effective polymer in retaining skin moisture for longer than untreated skin, water, propylene glycol, and native chitosan. Additionally, from the mangosteen extract deodorant creams with different H-CMCH concentrations (0.5–2.5% *w*/*v*), the appropriate H-CMCH content was selected from an accelerated stability test with six heating/cooling cycles. For the developed deodorant cream with 1.0% (*w*/*v*) H-CMCH, the viscosity and pH were unchanged after storage in the accelerated state, while the a* and b* values of the other formulas were slightly increased and the L* values was moderately decreased. Therefore, in deodorant cream development, 1.0%(*w*/*v*) H-CMCH was used for the optimal formula. Results indicated that the synergistic activity of ME and H-CMCH in emulsion creams had good potential as an effective skin moisturizing agent enhancer and good deodorizing activity against trans-2-nonenal odor, antioxidant properties, and antibacterial properties. Future studies may include investigation on modeling and numerical simulation of product stability. In addition, the engineering rheological properties of CMCH and creams should also be subsequently investigated.

## Figures and Tables

**Table 1:** Deodorant cream formulas with different content of H-CMCH (0.5, 1.0, 1.5, 2.0, and 2.5% (*w*/*v*)).

Ingredients	(% *w*/*v*)				
	H1 (0.5)	H2 (1.0)	H3 (1.5)	H4 (2.0)	H5 (2.5)
**Phase A (oil phase)**					
Aluminium chlorohydrate	40.0	40.0	40.0	40.0	40.0
Stearyl alcohol	2.0	2.0	2.0	2.0	2.0
Ceteareth-25	2.0	2.0	2.0	2.0	2.0
Glyceryl monostearate	2.0	2.0	2.0	2.0	2.0
Mineral oil	5.0	5.0	5.0	5.0	5.0
**Phase B (aqueous phase)**					
Glycerin	2.0	2.0	2.0	2.0	2.0
Propylene glycol	5.0	5.0	5.0	5.0	5.0
Distilled water	41.4	40.9	40.4	39.9	39.4
**Phase C**					
ME	0.1	0.1	0.1	0.1	0.1
**Phase D**					
H-CMCH	0.5	1.0	1.5	2.0	2.5
**Phase E**					
Perfume	0.01	0.01	0.01	0.01	0.01

**Table 2:** Antioxidant properties of the developed deodorant cream compared to prototype formula.

Samples	IC_50_ DPPH^ns^ (µg/mL)	IC_50_ ABTS (µg/mL)	FRAP (µmoL Fe^2+^/g Sample)
(1) Deodorant cream prototype (no ME and H-CMCH)	11.4 ± 2.8	12.7 ^a^ ± 1.0	47.5 ^b^ ± 0.4
(2) Developed deodorant cream (ME + 1% H-CMCH)	13.7 ± 3.0	7.7 ^b^ ± 2.8	51.8 ^a^ ± 0.6

Different letters indicate significant different between columns (*p* < 0.05).

**Table 3:** Inhibition zone of prototype deodorant cream and developed deodorant cream.

	Inhibition Zone (mm)					
Samples (10 mg/mL)	*S. aureus*	*S. epidermidis*	*Corynebacterium* spp.	*B. subtilis*	*P. aeruginosa*	*E. coli*
(1) Deodorant cream prototype (no ME and H-CMCH)	6.6 ^c^ ± 0.3	6.6 ^c^ ± 0.8	7.2 ^c^ ± 0.9	6.9 ^c^ ± 1.1	6.5 ^c^ ± 0.5	6.0 ^c^ ± 0.7
(2) Developed deodorant cream (ME + 1% H-CMCH)	13.3 ^b^ ± 0.6	19.2 ^b^ ± 1.2	25.3 ^b^ ± 0.5	21.7 ^b^ ± 2.1	7.9 ^b^ ± 0.8	12.4 ^b^ ± 1.2
(3) Streptomycin	19.3 ^a^ ± 0.5	27.2 ^a^ ± 0.4	36.8 ^a^ ± 1.1	30.0 ^a^ ± 1.9	10.6 ^a^ ± 0.5	19.5 ^a^ ± 0.5

Different letters indicate significant different between columns (*p* < 0.05).

## Data Availability

Not applicable.

## Supplementary Materials

The following are available online at https://www.mdpi.com/article/10.3390/polym14010178/s1. Figure S1. FT-IR spectra of (**a**) high Mw native chitosan; (**b**) H-CMCH; Figure S2. pH of deodorant creams adding H-CMCH by heating-cooling cycle for up to 6 cycles; Figure S3. Viscosity of deodorant creams adding H-CMCH by heating-cooling cycle for up to 6 cycles; Figure S4. Lightness (L*) of deodorant creams adding H-CMCH by heating-cooling cycle for up to 6 cycles; Figure S5. Red/green coordinate (a*) of deodorant creams adding H-CMCH by heating-cooling cycle for up to 6 cycles; Figure S6. Yellow/blue coordinate (b*) of deodorant creams adding H-CMCH by heating-cooling cycle for up to 6 cycles.
