# Supplementary material for: Synergistics of Carboxymethyl Chitosan and Mangosteen Extract as Enhancing Moisturizing, Antioxidant, Antibacterial, and Deodorizing Properties in Emulsion Cream
Source: Polymers (Basel). 2022 Jan 3;14(1):178. doi: 10.3390/polym14010178 (PMC8747190; doi:10.3390/polym14010178)
Supplement: Supplementary file 1 [file polymers-14-00178-s001.zip › polymers-1485653-supplementary.pdf]

Supplementary Data

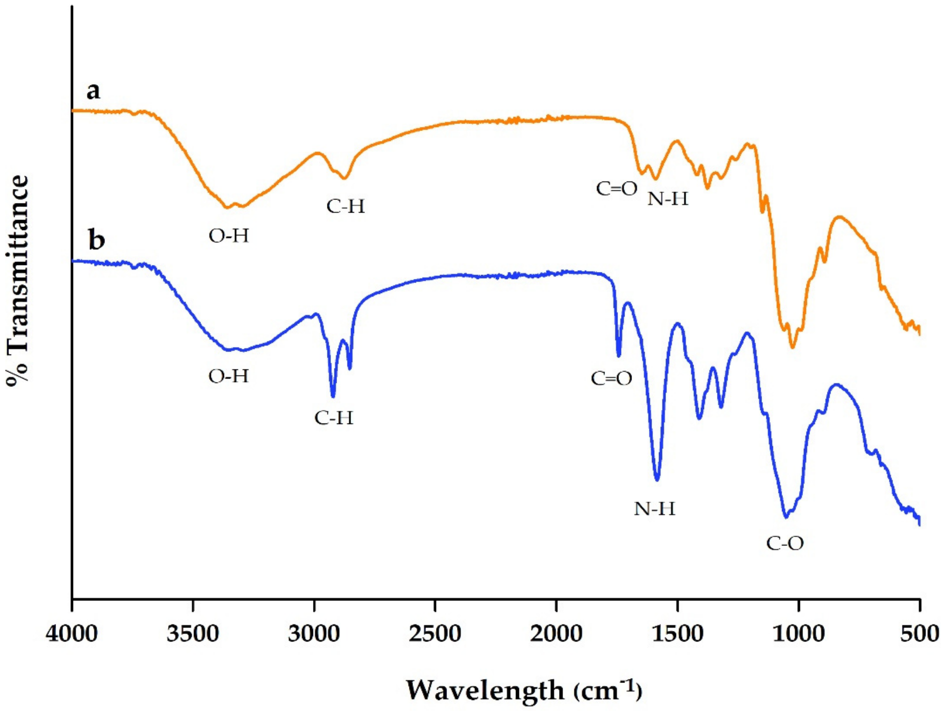

Figure S1. FT-IR spectra of (**a**) high Mw native chitosan; (**b**) H-CMCH.

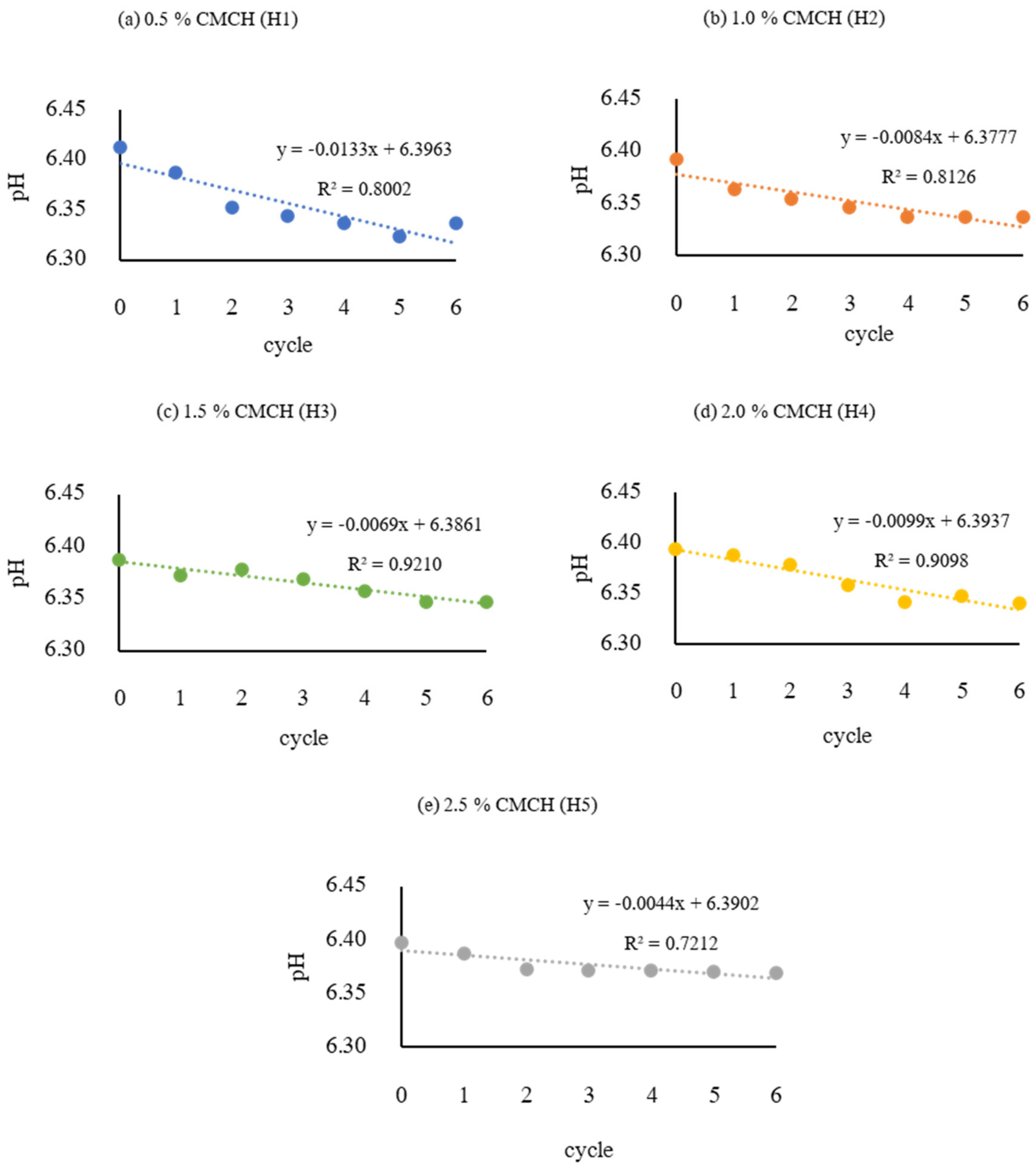

Figure S2. pH of deodorant creams adding H-CMCH by heating-cooling cycle for up to 6 cycles.

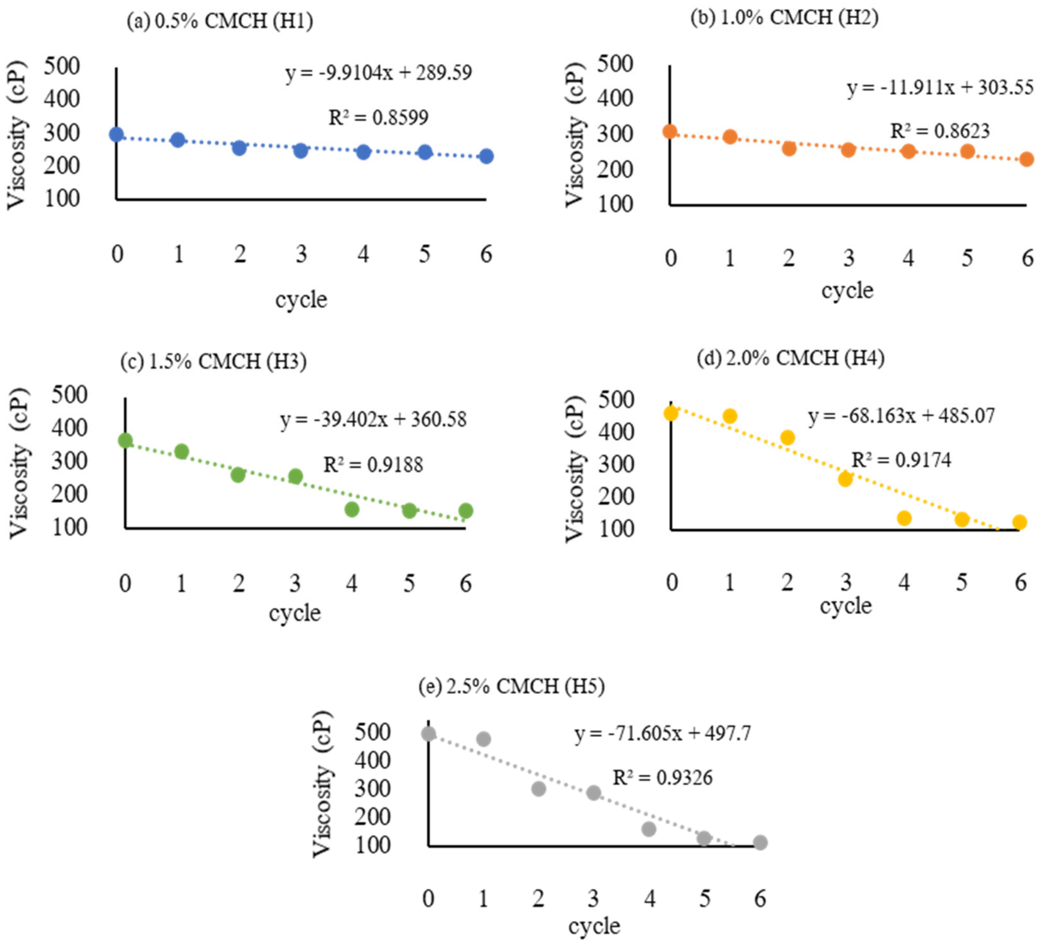

Figure S3. Viscosity of deodorant creams adding H-CMCH by heating-cooling cycle for up to 6 cycles.

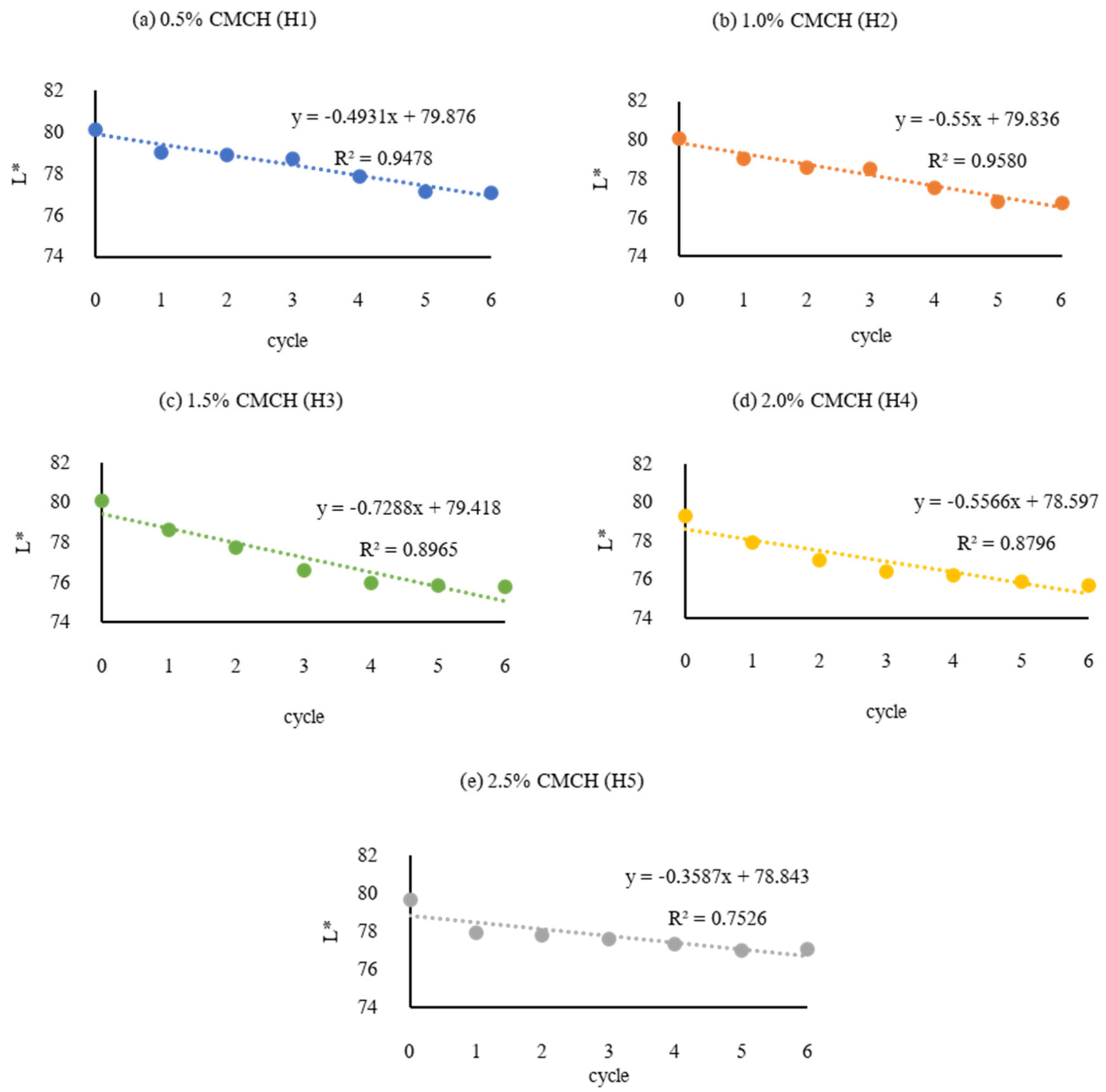

Figure S4. Lightness (L*) of deodorant creams adding H-CMCH by heating-cooling cycle for up to 6 cycles.

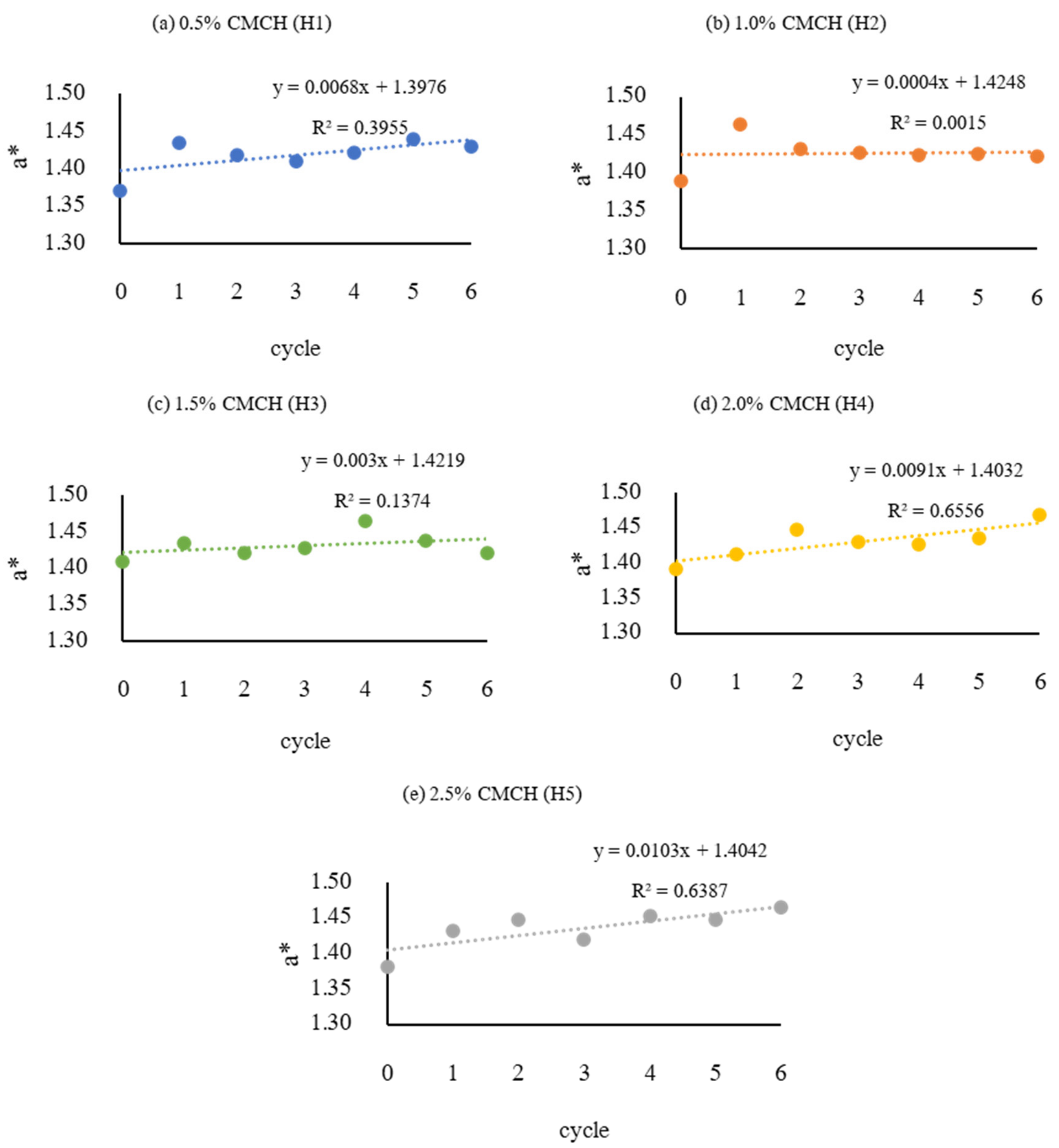

Figure S5. Red/green coordinate (a*) of deodorant creams adding H-CMCH by heating-cooling cycle for up to 6 cycles.

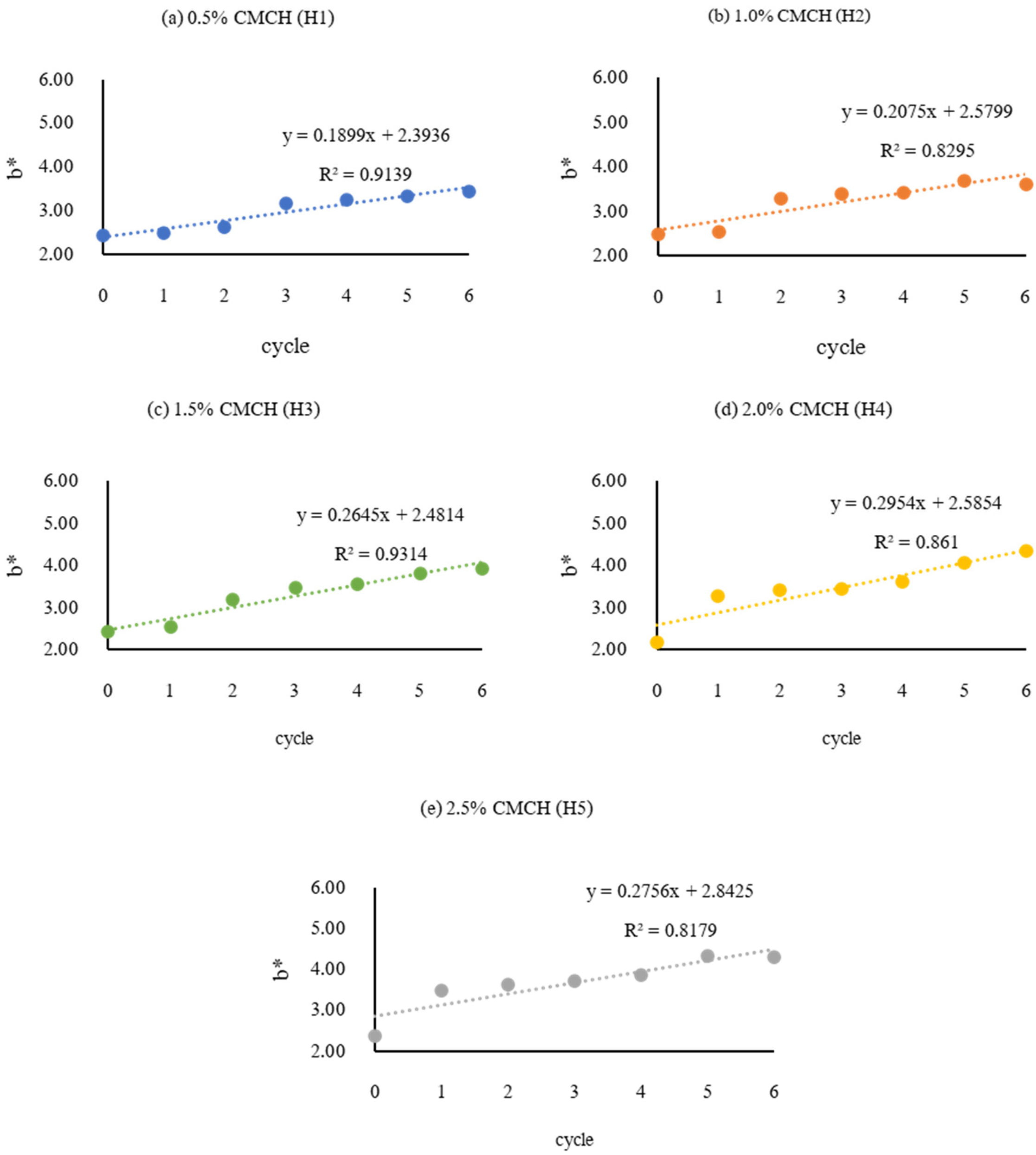

Figure S6. Yellow/blue coordinate (b*) of deodorant creams adding H-CMCH by heating-cooling cycle for up to 6 cycles.
